# Correlation between macular changes and the peripapillary nerve fiber layer in primary open angle glaucoma

**Published:** 2014-03-25

**Authors:** D Manasia, L Voinea, ID Vasinca, C Alexandrescu

**Affiliations:** * "Titu Maiorescu" University of Medicine Bucharest; ** "Carol Davila" University of Medicine and Pharmacy, Bucharest

**Keywords:** macula, glaucoma, RNFL, optical coherence tomography

## Abstract

Glaucoma is a leading cause of blindness and the early diagnosis is crucial for treatment and follow-up in the progression of the disease.

Objective: To evaluate the changes in mean macular thickness and volume and compare them with the mean thickness of the peripapillary nerve fiber layer in primary open angle glaucoma, using Time Domain (TD) optical coherence tomography ( OCT).

Method: The examinations were conducted on 275 eyes of 138 patients, as it follows: 203 eyes of 102 patients diagnosed with primary open angle glaucoma in various stages of evolution, representing the study group, and 72 normal eyes from 36 patients, representing the control group. The study was conducted from March 2010 to December 2012. All the patients gave their consent, in accordance with the Helsinki Declaration.

The study group showed a decrease in mean macular thickness and volume, as well as mean thickness of the peripapillary nerve fiber layer (RNFL) compared to the control group.

According to the OCT measurements, the results of the study have shown that the decrease of the mean thickness of the RNFL is a better differentiator between glaucomatous and normal eyes compared to the decrease of the mean macular thickness and volume.

## Introduction

Open-angle glaucoma is defined as a “multifactorial optic neuropathy with an acquired atrophy of the optic nerve and loss of retinal ganglion cells and their axons, developing in the presence of open anterior chamber angles, and manifesting characteristic visual field abnormalities" [**1**]. The optic nerve is composed of retinal ganglion cell axons. The approximately 1 million axons forming the optic nerve form the retinian nervous fiber layer and converge towards the optic papilla [**2**]. The nervous fiber layer is thicker in the peripapillary area compared to the retinian periphery and defects in this layer are often a sign of glaucomatous affection [**3**]. These defects can precede modifications of the visual field [**4**]. Studies on primates and mice suggest that high intraocular pressure might be the determining factor of the cellular events that trigger apoptosis of the ganglion cells [**5**]. In the macula there are up to 10 rows of ganglion cells, with the largest concentration in the parafoveal region [**6**]. The ganglion cells layer together with the nerve fiber layer represent approximately 30-45% of the macular thickness [**7**]. The ganglion cell loss in glaucoma leads to the reduction of the thickness and, implicitly, of the macular volume and this has been shown by numerous studies. 

 OCT is a non-contact, non invasive imaging method that uses near-infrared light to scan the retina and optic disc and has become an important component of the retina and optic nerve examination [**8**]. By using OCT we can obtain high-resolution cross-sectional images of the macula, RNFL and optic papilla [4,9].

## Material and method

 Mean macular thickness and volume, mean RNFL thickness with OCT and mean defect (MD) with perimeter were measured for 275 eyes, 72 normal and 203 eyes with glaucoma. Based on changes in the perimeter, the study group was divided into one group of glaucoma suspects and patients with preperimetric open angle glaucoma (GSPP) which comprised 78 eyes, and another group of patients with perimetric open angle glaucoma (GP) comprising 125 eyes. The patients were examined upon check-control protocol, and all the investigations for one patient were done on the same day. The investigations included: examination of the visual acuity and refraction, measurement of the intraocular pressure using Goldmann aplanometry, pachymetry, gonioscopy, visual field test with the Octopus 1-2-3 perimeter, OCT for the macula and the optic nerve, using a Zeiss Stratus TD- OCT, software 5.01. The measurements of macular thickness and volume were done by “fast macular thickness scan" and the measurement of the thickness of the peripapillary nerve fiber layer was done by "Fast RNFL thickness 3.4". 

 Study group selection 

 The lots comprised patients between 40 and 78 years old, irrespective of gender. Patients with visual acuity under 20/40 with best correction were excluded, as well as patients with refraction errors: hypermetropia with over +3 spherical diopters, myopia with over -5 spherical diopters, astigmatism with over 2 cylindrical diopters, patients with affections that might affect the macula or the optic nerve, such as: diabetic retinopathy, macular degeneration, vascular occlusions, neuropathies. Patients who had undergone eye surgery – except for those with pseudofac implants with visual acuity within the set limits – and patients who had undergone chronical corticoid treatment, ocular or general, were also excluded. 

 Measurements of macular thickness, macular volume and nervous fiber layer 

 Measurements were done with the ocular tomograph STRATUS OCT, software version 5.0.1. 

 For the macular measurements the scanning method “Fast Macular Thickness" was utilized; this method measures the macular thickness and volume of a 6mm diameter area, centered on the fovea, area subdivided into 9 zones by three circles at 1, 3 and 6 mm from the fovea. The scan is performed on six radial lines, which intersect at the fovea, and each scan line is comprised of 128 individual scans [**10**]. By protocol analysis, macular thickness is measured in microns and macular volume in microns cubed for each of the nine areas [**8**]. 

 For the measurements of the thickness of the nervous fiber layer, the scanning method of Fast RNFL Thickness (3.4) was utilized; through this method, three circular scans are performed on a 3,46mm diameter circle centered on the optic nerve, with a total of 768 scans. The scanning performs a quantitative evaluation of the RNFL in the peripapillary area and RNFL thickness is measured also in microns [**8**]. 

 OCT provides values for the entire scanned area, by sectors and quadrants, as well as comparisons with the normative database. 

 Statistical methods 

 The statistical analysis was done with the statistic programs R, version 2.15.3, and the statistic, versions 10 and 12. To determine whether the macular thickness and volume differ among normal patients, glaucoma suspects and patients with glaucomatous eyes, statistical tests Chi-square, P and the Pearson correlation were used. Dispersion graphs were also used in the data analysis. 

## Results

 Results of the OCT measurements for the mean macular thickness: 

 For the N group, the mean value was 246,88 microns, for the GSPP group the mean value was of 236,33 microns and for the GP group, the mean value was 221,30 microns . The mean values of the macular thickness for the three groups are illustrated in Graph 1 below. 

**Fig. 1:**
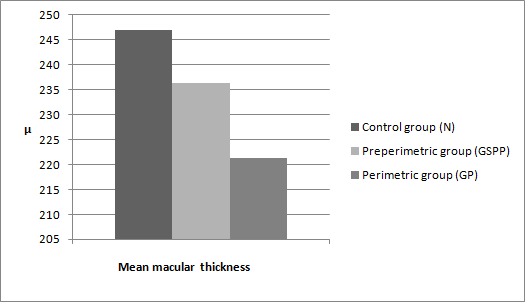
Mean macular thickness in the three groups. N represents the control group, GSPP the group of glaucoma suspects and preperimetric glaucoma patients and GP represents the group of patients with perimetric glaucoma.µ represents microns

 Results of the OCT measurements for the mean macular volume 

 In the control group, the macular volume had a mean value of 6,789microns cubed, the GSPP group had a mean value of 6,451 microns cubed and in the GP group, the mean value was of 6,017 microns cubed .The mean values of macular volume for the three groups are shown in graph 2 below. 

**Fig. 2:**
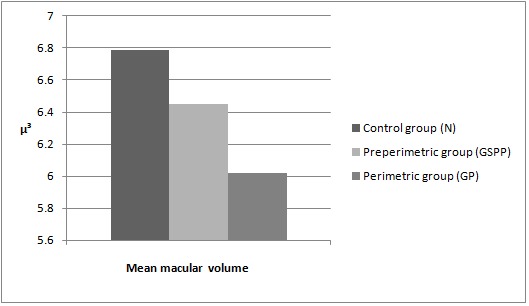
Mean macular volume in the three groups. N represents the control group, GSPP the group of glaucoma suspects and preperimetric glaucoma patients and GP represents the group of patients with perimetric glaucoma.µ³ represents microns cubed

 Results of the OCT measurements for the mean thickness of the papillary nerve fiber layer (RNFL) 

 In group N, the mean RNFL thickness was of 100,04 microns, in the GSPP group the mean value was of 83,99 microns, and in the GP group the mean thickness was of 64,13 microns. The results are shown in Graph 3 below. 

**Fig. 3:**
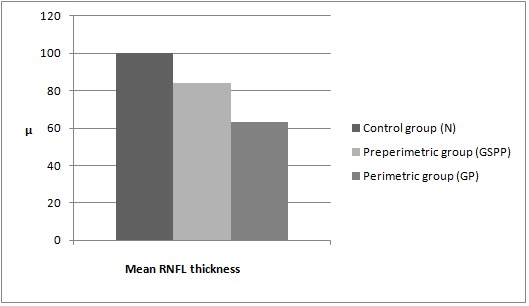
Mean RNFL thickness in the three studied groups: N, GSPP and GP.µ represents microns

 Results by studied group 

 In group N, the measurements were as it follows: for the mean macular thickness, the mean was 246,88 microns with a minimum of 237,22 microns and maximum of 268 microns, the mean macular volume was of 6,789 microns cubed, the minimum was 6,505 microns cubed and the maximum 7,185 microns cubed, and the mean RNFL thickness was 100,04 microns with a minimum of 88,82 microns and a maximum of 113,53 microns. The results are summarized in the table below. 

**Table 1:** Values of the OCT measurements for the mean macular thickness, mean macular volume and mean peripapillary RNFL thickness for the control group.

Values	Minimum	Median	Maximum
Mean macular thickness (microns)	237,22	246,88	268
Mean macular volume (microns cubed)	6,505	6,789	7,185
Mean peripapillary RNFL (microns)	88,82	100,04	113,53

 In the GSPP group, the values for the mean macular thickness were the following: a 236,33 microns mean with a 223,77 microns minimum and a 256,44 microns maximum, and for the mean macular volume the mean was 6,451 microns cubed with a minimum of 6,156 microns cubed and a maximum of 6,960 microns cubed, and for the mean peripapillary RNFL thickness the mean was 83,94 microns, the minimum was 73,44 microns and the maximum was 103,09 microns. The results are summarized in Table 2 below. 

**Table 2:** Values of the OCT measurements for the mean macular thickness, mean macular volume and mean peripapillary RNFL thickness for glaucoma suspects and patients with preperimetric glaucoma

Values	Minimum	Median	Maximum
Mean macular thickness (microns)	223,77	236,33	256,44
Mean macular volume (microns cubed)	6,156	6,451	6,960
Mean peripapillary RNFL (microns)	73,44	83,94	103,09

For the group of perimetric glaucoma patients, the OCT measurements generated the following values: for the mean macular thickness, the mean was of 221,30 microns, with a minimum of 189,66 microns and a maximum of 246,44 microns; for the mean macular volume, the mean was of 6,017 microns cubed, with a minimum of 5,173 microns cubed and a maximum of 6,662 microns cubed and for the mean thickness of peripapillary RNFL the mean was 63,43 microns with a minimum of 37,89 microns and a maximum of 79,40 microns. The results are summarized in Table 3. 

**Table 3:** Values of the OCT measurements for the mean macular thickness, mean macular volume and mean peripapillary RNFL thickness for patients with perimetric glaucoma.

Values	Minimum	Median	Maximum
Mean macular thickness (microns)	189,66	221,30	246,44
Mean macular volume (microns cubed)	5,173	6,017	6,662
Mean peripapillary RNFL (microns)	37,89	63,43	79,40

 Statistical analysis

 The results, measured with a confidence level of 95%, show that the macular thickness and volume, and especially RNFL are significantly lower in glaucomatous eyes (Chi-square >3.841 and p<0,05 indicate results that are statistically significant). The results also show a statistically significant higher correlation between RNFL and MD in glaucomatous eyes compared with the correlation between macular thickness and MD. The correlation between macular volume and MD was the lowest. 

 Compared to the control group, the macular thickness decreases on average by 4.27% in the group of glaucoma suspects and preperimetric glaucoma patients, and the volume is on average 5% lower. In the glaucomatous patients’’ group the macular thickness is on average 10.36% lower compared to the control group, while the macular volume is 11.37% lower. This decrease of macular thickness and volume in the GP group is statistically more significant than in the GSPP group, Chi-square is 8.67, significantly higher than 3.841, which represents the minimum Chi-square value for statistically significant results; also, the statistical parameter p < 0.05, indicating statistically significant results. The decrease in volume in group GP is also statistically significant compared to group GSPP, with Chi-square of 8.18 and p<0.05. The greater decrease of thickness in glaucomatous eyes is also reflected in the dispersion graph below, which represents the macular thickness and the volume in the three studied groups. 

**Fig. 4:**
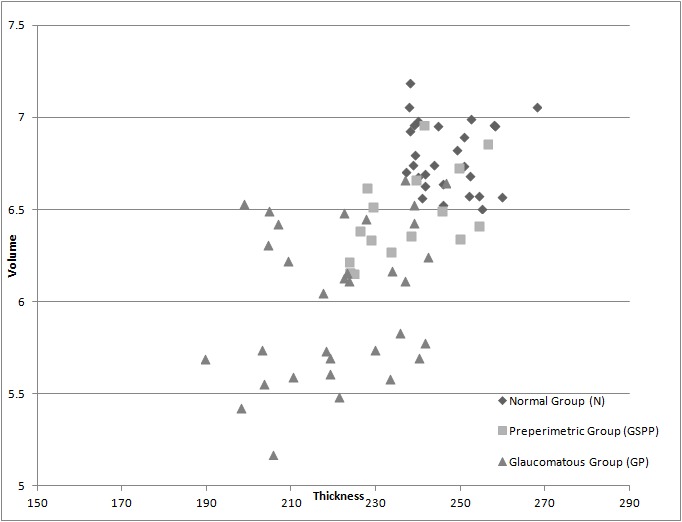
Shows the dispersion of macular thickness and volume in the three studied groups

 Compared to the control group, RNFL is 16% lower in the GSPP group and 37% lower in the GP group. The decrease in RNFL is more correlated with the glaucoma throughout all the stages of the disease compared to the decrease in macular thickness. For RNFL the Chi-square is of 26.14, significantly higher than the necessary minimum of 3.841, and also higher than the Chi-square obtained when analyzing the macular thickness, which was 8.67. These results are statistically significant, with p<0.05. 

 The results of the measurements for the macular thickness and the RNFL thickness are shown in the dispersion graph below. 

**Fig. 5:**
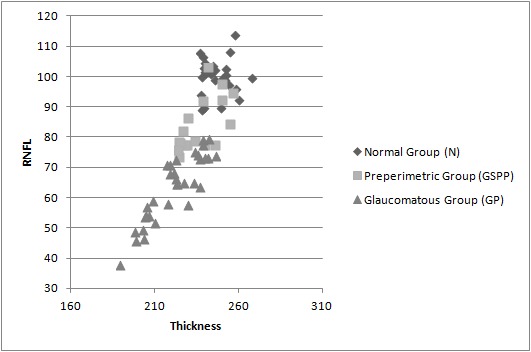
Shows the macular thickness and RNFL thickness for the three studied groups

 Correlation macula-RNFL 

 The results show a strong correlation between the stage of glaucoma and the decrease in RNFL thickness and macular thickness for the group of glaucomatous eyes. The decrease in volume shows a lower correlation. The glaucomatous eyes group is more correlated with glaucoma than the GSPP and control group. Correlation between macular thickness and MD for the glaucomatous eyes is -0.84, while for the GSPP and control groups the correlation is -0.13 and -0.18 respectively. The stronger correlation for glaucomatous eyes is even more evident for RNFL and MD, with a -0.96 correlation for glaucomatous eyes, a -0.30 correlation for GSPP and -0.24 for the normal eyes. Macular volume shows a lower correlation with the advance of glaucoma, with a correlation of -0.22 for the glaucomatous eyes. 

## Discussion

 OCT allows the measurement of macular thickness and volume and the thickness of the peripapillary nervous fiber layer in normal and glaucomatous eyes. 

 From the data obtained in this study we can conclude that in glaucomatous eyes the mean macular thickness and volume decrease significantly compared to normal eyes, and this has also been proven in similar studies [**7,10-14**]. The macular thickness and volume are significantly lower in glaucoma. Decrease in macular volume is correlated with the decrease in macular thickness. The decrease is more significant in eyes with more advanced glaucoma compared to glaucoma suspects and patients with preperimetric glaucoma: chi-square is 8.67 and p<0,05 for the macular thickness, while for the macular volume chi-square is 8.18 and p<0,05, values that are statistically significant. Measurements of the RNFL showed a significant decrease, similar to many other studies [**15,16**]. The decrease in thickness of the peripapillary nerve fiber layer is stronger correlated with glaucoma for all the phases of glaucoma evolution, compared to the decrease in macular thickness. Also, for the RNFL chi-square is 26.14, statistically significant (p<0,05), greater than the chi-square values obtained for the mean macular thickness and volume. The results are in line with other studies [**17-21**]. Also, the correlation between RNFL and the stage of glaucoma is the most significant, of -0.96, showing that the higher MD becomes, the lower the thickness of RNFL, meaning that as glaucoma becomes more advanced, the sharper the decrease in thickness for the RNFL. This relation is also holds for the stage of glaucoma and macular thickness, although not as strong as the RNFL, showing a correlation of -0.84. Correlations this close to -1 show a significantly strong linear correlation. Volume shows a lower correlation of -0.22. Thus, RNFL shows the strongest correlation with glaucoma, followed by macular thickness and macular volume. 

## Conclusions

 Using the OCT Stratus time domain we can detect changes in the macular thickness, macular volume and retinian nerve fiber layer thickness parameters. All these parameters are significantly decreased compared to normal eyes, even in incipient stages of glaucoma when the visual field changes are not yet detectable. The decrease in RNFL thickness is the parameter most correlated statistically with glaucoma. 
